# Renal Failure Impact on the Outcomes of ST-Segment Elevation Myocardial Infarction Patients Due to a Left Main Coronary Culprit Lesion Treated Using a Primary Percutaneous Coronary Intervention

**DOI:** 10.3390/jcm8040565

**Published:** 2019-04-25

**Authors:** Cãlin Homorodean, Adrian Corneliu Iancu, Ioana Mihaela Dregoesc, Mihai Spînu, Mihai Claudiu Ober, Dan Tãtaru, Daniel Leucuţa, Maria Olinic, Dan Mircea Olinic

**Affiliations:** 1 1st Dept. of Internal Medicine, “Iuliu Hatieganu ” University of Medicine and Pharmacy, 4000060 Cluj-Napoca, Romania; chomorodean@yahoo.com (C.H.); spinu_mihai@yahoo.com (M.S.); tataru.cardio@gmail.com (D.T.); maria_olinic@yahoo.com (M.O.); dolinic@yahoo.com (D.M.O.); 2 Emergency County Hospital, 400000 Cluj-Napoca, Romania; mihai_ober@yahoo.com; 3 Department of Cardiology, “Niculae Stãncioiu” Heart Institute, “Iuliu Hatieganu ” University of Medicine and Pharmacy, 400001 Cluj-Napoca, Romania; adrian_iancu@hotmail.com; 4 Dept. of Medical Informatics and Biostatistics, “Iuliu Hatieganu ” University of Medicine and Pharmacy, 400349 Cluj-Napoca, Romania; danny.ldc@gmail.com

**Keywords:** left main coronary artery, ST-elevation myocardial infarction, primary percutaneous coronary intervention, renal failure, SYNTAX Score

## Abstract

Background: Patients with ST-segment elevation myocardial infarction (STEMI) and primary percutaneous coronary intervention (PPCI) on a left main culprit lesion have very high mortality rates. The interaction of chronic kidney disease (CKD) with such a catastrophic acute event on the background of their highly complex atherosclerotic lesions is not well established. Therefore, we sought to evaluate in these patients the influence of the estimated glomerular filtration rate (eGFR) on short- and long-term mortality. Methods: We retrospectively analyzed renal function in 81 patients with STEMI and PPCI on a left main culprit lesion from two tertiary centers. Results: Patients were divided in two groups according to an eGFR cut-off of 60 mL/min/1.73 m^2^: 40 patients with CKD and 41 without CKD. Patients with renal failure were older, had more diabetes, and had experienced more frequent myocardial infarction MIs. CKD patients had a higher baseline-SYNTAX score (*p* = 0.015), higher residual-SYNTAX score (*p* < 0.001), and lower SYNTAX revascularization index-SRI (*p* = 0.003). Mortality at 30-day, 1-year, and 3-year follow-ups were not significantly different between the two groups. However, when analyzed as a continuous variable, eGFR emerged as a predictor of 1-year mortality, both in univariate analysis (OR = 0.97, 95% CI: 0.95–0.99, *p* = 0.005) and in multivariate analysis, after adjusting for cardiogenic shock and Thrombolysis in Myocardial Infarction TIMI 0/1 flow (OR = 0.975, 95% CI: 0.95–0.99, *p* = 0.021). Conclusions: In STEMI with PPCI on a left main culprit lesion, renal failure was associated with more complex coronary lesions and less complete revascularization, and turned out to be an independent predictor of mortality at 1-year follow-up.

## 1. Introduction

Chronic kidney disease (CKD) is a strong risk factor for fatal and nonfatal cardiovascular events [1,2,3]. Even mildly decreased kidney function with an estimated glomerular filtration rate (eGFR) of 60–90 mL/min/1.73 m^2^ independently predicts long-term mortality, both in coronary stable [4] and ST-segment elevation myocardial infarction (STEMI) patients [5]. There are data showing that renal dysfunction per se might initiate and accelerate adverse cardiac events [4].

Revascularization improves the poor outcomes in CKD patients with coronary artery disease (CAD). Although the percutaneous coronary intervention (PCI) procedure itself is challenging and associated with an upfront risk of further kidney injury or procedural complications [6], it actually decreases long-term mortality [6].

Patients with STEMI due to an unprotected left main coronary artery (UPLMCA) lesion are particular in sharing both a catastrophic acute instability and a very high atherosclerothic burden, quantified by baseline SYNTAX (synergy between PCI with TAXUS™ and cardiac surgery) score [7]. Survivals of an acute UPLMCA occlusion treated using PCI have a damaged myocardium and residual coronary lesions (residual SYNTAX score) consistent with the completeness of revascularization. Therefore, they are vulnerable to residual ischemia or future acute events. These vulnerabilities are supposed to be oversized in patients with renal failure because of a more extensive and complex CAD [8] and more vulnerable non-culprit plaques [3,9] than in patients with normal renal function. What is more, in the CKD group, the myocardial substrate stroked by the index acute left main event might already be damaged by the increased fibrosis [3].

Although there are data from randomized trials on the outcomes of CKD patients with stable left main disease [10], the situation is different regarding STEMI patients due to UPLMCA with renal impairment, where data are few and conflicting with gaps in the evidence [11,12,13,14]. Some of these studies [11,13] used creatinine levels to characterize renal function, which is known as an unreliable method when compared to the eGFR.

Therefore, we sought to retrospectively evaluate the influence of eGFR, calculated using the most currently accepted equation (CKD Epidemiology Collaboration) on short- and long-term mortality of the patients with STEMI due to UPLMCA treated using primary PCI.

## 2. Materials and Methods

This was a retrospective observational cohort study. Patients with STEMI and primary PCI for a UPLMCA culprit lesion were identified in the registries of two centers in Cluj-Napoca, Romania. These two centers perform more than 3000 PCI procedures annually, of which 900 are primary PCIs, in the setting of acute STEMI. Eight senior operators were involved in the study, each performing more than 250 interventions annually, 15–50 of these being left main PCI procedures. The inclusion period was between January 2010 and March 2017.

The inclusion criteria, presented in detail within a previous paper [7], were ongoing ischemic chest pain with a duration of more than 30 min, accompanied by ST-segment elevation of at least 0.2 mV in two contiguous electrocardiographic (ECG) leads, left main STEMI equivalent ECG changes [15], new left bundle branch block, and/or cardiogenic shock.

Coronary flow was graded according to the Thrombolysis in Myocardial Infarction TIMI classification system. Collateral flow was evaluated using Rentrop criteria [16].

Left main was considered “unprotected” in the absence of any patent left coronary artery bypass grafts.

UPLMCA was considered the culprit vessel in the case of a more than 90% stenosis or in the case of an angiographic complicated lesion: dissection, thrombus, plaque rupture, or TIMI 0–2 flow.

Baseline SYNTAX score I (synergy between PCI with TAXUS™ and cardiac surgery), baseline SYNTAX score II, and residual SYNTAX score were calculated for each patient by two independent senior interventional cardiologists. SYNTAX score revascularization index (SRI) represents the proportion of CAD burden treated using PCI [17]. It was calculated using the formula [17]:

SRI = (1−(rSS/bSS)) × 100 rSS = residual SYNTAX Score bSS = baseline SYNTAX Score

Technical success was defined as less than 30% residual stenosis in the presence of TIMI 3 flow.

### 2.1. Baseline Blood Investigations

Baseline serum creatinine was derived from the venous sample acquired in the emergency ward prior to cath-lab admission. The estimated glomerular filtration rate (eGFR) was calculated using the CKD Epidemiology Collaboration (CKD-EPI) equation, as per the Kidney Disease: Improving Global Outcomes (KDIGO) guidelines [18]. CKD was defined as an estimated glomerular filtration rate (eGFR) < 60 mL/min/1.73 m^2^. Patients were separated into two groups: CKD with GFR < 60 mL/min and non-CKD with a GFR > 60 mL/min. Urine output was monitored before and after the primary PCI procedure, defining oliguria as an output less than 0.5 mL/kg/min.

All-cause mortality was the primary end-point of the study. Mortality data were obtained from medical records for inpatient deaths. Regarding end-point after discharge, information was obtained from primary care physician records, telephone interviews, questionnaires sent by mail, or domicile visits. Data on the vital status was available in the electronic records of the national assurance company in all patients. Medical records of the follow-up events were obtained in 90% of the patients. All-cause mortality was reported according to Academic Research Consortium recommendations [19]. Follow-up ended on 31 December 2017.

### 2.2. Statistical Analysis

Normally distributed continuous variables (e.g., age) were presented as mean ± standard deviation and the significance between independent groups was tested using the independent samples *t*-test. Skewed continuous variables (e.g., bSYNTAX, rSYNTAX scores) were presented as median (interquartile range) and were analyzed using the Mann–Whitney U test. Categorical variables were presented as counts and proportions, and for statistical comparisons we used the chi-square or the Fisher’s exact tests. Normality in variable distributions was tested using the Shapiro–Wilk test. Correlations between continuous variables were assessed using the partial Spearman correlation coefficient, with adjustment for age, diabetes, left ventricular ejection fraction less than 30%, and prior myocardial infarction (MI) with the associated statistical test.

To assess the relationship between variables of interest and mortality, logistic regressions were used. Unadjusted models were built first, followed by models that included the variables of interest. Two models were developed: one adjusted for the presence of shock and TIMI flow 2/3 versus 0/1 and a second one adjusted for left ventricle low ejection fraction (<30%), diabetes mellitus, and prior MI. The odds ratio along with 95% confidence intervals and *p*-values were computed for each regression. The goodness-of-fit, the presence of multicollinearity, and misspecification were checked for each model. The log-linearity assumption was checked for continuous variables.

A two-sided *p*-value < 0.05 was considered statistically significant.

Statistical analysis was performed using the R environment for statistical computing and graphics (R Foundation for Statistical Computing, Vienna, Austria), version 3.4.4.

## 3. Results

Baseline renal function was evaluated in 81 patients with STEMI due to UPLMCA whose outcomes were presented in a previous paper [7]. Among the 30-day survivors, mean follow-up was 36 months.

At presentation, the mean eGFR was 63.03 ± 28.7 mL/min/1.73 m^2^. The distribution of baseline eGFR was roughly normal. Among the 81 enrolled patients, 40 were classified into a CKD group with a mean eGFR of 38.75 ± 13.97 mL/min/1.73 m^2^ and 41 into a non-CKD group. All patients had preserved urine output before the PCI procedure.

There were significant differences between the two groups of patients. Baseline characteristics are reported in Table 1. CKD patients were older (*p* < 0.001), were more likely to have diabetes (*p* = 0.035), were more likely to have a history of myocardial infarction (*p* = 0.002), and had a lower ejection fraction (*p* = 0.005) than non-CKD patients.

Angiographic and procedural characteristics are reported in Table 2. Patients with CKD had a higher baseline-SYNTAX score (median of 30.5 vs. 23, *p* = 0.015), higher residual-SYNTAX score (8.5 vs. 0, *p* < 0.001), lower SRI (67.8 vs. 100, *p* = 0.003), and were more likely to have multivessel coronary artery disease (87.5% vs. 51.2%, *p* < 0.001) than non-CKD ones. eGFR was inversely correlated (after partial adjustment for age, diabetes, left ventricular ejection fraction <30%, prior myocardial infarction) with bSYNTAX score (r = −0.28, *p* = 0.013) and rSYNTAX (r = −0.38, *p* < 0.001), and directly correlated with SRI (r = 0.38, *p* < 0.001).

### Effect of Low eGFR on Mortality

There were non-significant differences between CKD and non-CKD groups regarding 30-day (35% vs. 28.6%, *p* = 0.42), 1-year (55% vs. 34.14%, *p* = 0.059), and long-term mortalities (62.5% vs. 46.3%, *p* = 0.14). When performing a binary logistic regression, eGFR emerged as a predictor of cardiogenic shock (OR = 0.974, 95% CI = 0.95–0.99, *p* = 0.004).

Only one non-fatal MI was recorded during the first year of follow-up in a non-CKD patient.

In univariate analysis, eGFR was not significantly associated with 30-day mortality. Nevertheless, it became a mortality predictor at the 1-year (OR = 0.97, 95% CI = 0.95–0.99, *p* = 0.005) and 3-year follow-ups (OR = 0.98, 95% CI = 0.96–0.99, *p* = 0.016) (Table 3).

In multivariate analysis, when adjusted for cardiogenic shock and TIMI 0/1 flow (Table 3), eGFR remained an independent predictor of 1-year mortality (OR = 0.975, 95% CI = 0.95–0.99, *p* = 0.021) but not at the 3-year follow-up (OR = 0.98, 95% CI = 0.96–1.001, *p* = 0.063).

In another mortality prediction model, we included eGFR, diabetes, antecedents of MI, and ejection fraction. eGFR emerged as an independent mortality predictor at the 1-year follow-up (OR = 0.97, 95% CI = 0.95–0.99, *p* = 0.027) (Table 4).

## 4. Discussion

In patients with STEMI due to the UPLMCA culprit vessel being treated using primary PCI, the main findings on the influence of renal dysfunction on short- and long-term outcomes are summarized below. When comparing the groups with and without CKD, as defined by the standard cut-off eGFR of 60 mL/min/1.73 m^2^, there were no differences in short- and long-term mortalities. However, when renal function was analyzed as a continuous eGFR variable, it turned out to be an independent predictor of mortality at the 1-year follow-up, even when adjusted for cardiogenic shock and TIMI 0/1 flow. This means that progressively worse renal impairment was associated with steadily increasing all-cause mortality rates.

Our analysis offers some data on the interplay between renal dysfunction and mortality.

Of the patients with STEMI due to UPLMCA, the CKD subgroup was more severely diseased, presenting with a higher lesion extent and complexity, as it is shown from their b-SYNTAX scores, which increase progressively with decreasing renal function (eGFR). The results of our study confirm those from post-mortem [8,20] and SYNTAX score studies [21] and extend them to these very acute patients with STEMI due to UPLMCA.

Following primary PCI, patients with renal failure had a higher r-SYNTAX score and lower SRIs as a consequence of less complete revascularization, most probably due to a propensity for complex and heavily calcified lesions. More residual lesions lead to poor outcomes since it is established that a greater CAD burden confers a significantly higher risk for clinical plaque progression [22]. Moreover, it has also been shown that patients with multivessel CAD during the original PCI were more likely to require non-target lesion PCI [22,23].

Plaques are more prone to rupture due to increased inflammation in CKD [3,9]. Optical coherence tomography (OCT) is one of the best imaging techniques for visualizing vulnerable plaques [24]. OCT studies revealed that a lower eGFR was associated with a larger lipid core in non-culprit plaques [9,25]. Additionally, calcium deposits damage vascular smooth muscle, impair vascular reactivity, and increase plaque rupture [1]. However, we did not observe an increase in nonfatal MI. It is likely that such events add to the outcomes as deaths and not non-fatal MI. This is in line with a previous study showing an increase in risk of cardiovascular death at 1 year without a concomitant increase in risk of MI [1].

CKD patients might develop acute heart failure easier, when LM occludes, due to concerted myocardial damages: fibrosis, coronary microcirculatory dysfunction [3], increased microvascular and obstruction after primary PCI [26]. Our data showed a correlation between eGFR and cardiogenic shock.

Evaluation of kidney function in our retrospective study has several shortcomings. We deemed it useful to quantify renal impairment in our patients as it is an important predictor of outcome in the settings of both acute and chronic kidney diseases [27].

The main issue is the differential diagnosis between impaired kidney function due to CKD versus acute kidney injury (AKI). Detection of renal impairment in STEMI patients at the moment of hospital admission may reflect a combination of acute hemodynamic instability as well as chronic renal disease, and therefore affect both short- and long-term risk [27]. In the absence of previous data on renal function, the probability of CKD versus AKI is to be inferred based on the clinical context. In our particular case, creatinine was assessed at presentation in the first few hours after onset of pain in patients. Specifically, in cardiogenic shock patients of our cohort, the mean delay between pain onset and presentation to emergency ward was 6.3 h. This short time of clinically evident acute illness is generally insufficient to a generate measurable increase in serum creatinine in most cases after acute kidney damage. In a large study on hypotensive patients, the average acute kidney injury onset time was 2.3 days after intensive care unit admission [28]. It is known that serum creatinine rise is commonly delayed after kidney function decline [29] and do not depict real-time changes in renal function. On the other hand, decrease in urine output is a more sensitive marker of AKI soon after onset of the risk factor for AKI due to the speed of the response [30]. Therefore, in this context, in patients with preserved urine output, impairment of kidney function was supposed to be chronic, and therefore evaluated via estimation of GFR according to KDIGO guidelines [18]. We also acknowledge that not all patients with mildly decreased GFR (grade 1 and 2) are certain to have CKD, as other markers of abnormalities of kidney structure or function persisting for >3 months were not documented in our cohort. Nevertheless, eGFR is an accurate measurement of kidney function, even in the absence of such markers of chronic kidney damage [30].

This is a common problem in trials evaluating the impact of renal insufficiency on the outcomes of patients with UPLMCA lesions, treated using PCI [10,11]. It is not a large population of patients and usually there are no previous records on the renal function [10]. It should be stressed that STEMI patients due to UPLMCA are an even smaller group as they account for roughly 2% of all primary PCIs [11]. Due to severe and extensive myocardial damage, the mortality rates are high, and some of the patients die before any medical contact.

However, regardless of the type of renal impairment, in our study, eGFR emerged as a predictor of one-year mortality independent of the cardiogenic shock.

There are studies suggesting that renal dysfunction is a good indicator of the overall systemic function and of the biological age, and therefore is not causative of mortality per se [5]. Actually, in our study, CKD patients were older, more likely to have diabetes or prior MI, and had a lower left ventricular ejection fraction. The significant association of eGFR with 1-year mortality does not necessary imply a causal relationship, although it turned out to be independent of diabetes, antecedents of MI, or ejection fraction. Previous studies had also shown an independent association with all-cause cardiac mortality, even after adjusting for cardiac risk factors [4], suggesting that renal dysfunction might initiate and accelerate adverse cardiac events [4].

Evidence from previous trials to provide information on the influence of low eGFR in STEMI patients due to UPLCA are scarce and conflicting, but rather negative [11,12,13,14]. In the largest registry to date, Patel et al [11] did not find renal dysfunction to be an independent short- or long-term mortality predictor in a large number of STEMI patients due to occluded left main. In a Korean multiregistry analysis [31], the risk of stent failure itself was similar between CKD and non-CKD patients.

### Limitations

First, the present study is an observational one, therefore it could not account for unmeasured confounders and causal inferences are difficult to make. Second, the number of patients is small. It could be easily increased if databases with an angiographic Digital Imaging and Communications in Medicine DICOM standardized structured reporting, similar to those developed by our group in echocardiography [32,33] would be developed and largely available. Third, the problem of differential diagnosis between CKD and AKI is a major limitation. It must be emphasized that serum creatinine was measured on admission and this might have led to the inclusion of individuals with acute kidney injury, whose eGFR was not estimated at equilibrium. However, this is an improbable major selection bias since the time from symptoms’ onset to probe collection was limited to a few hours and all included patients had preserved urinary output before primary PCI procedure. Fourth, due to the low event per variable ratio, we could not analyze all the variables from the two models into a single one without too much overfitting.

## 5. Conclusions

In patients with STEMI due to UPLMCA lesions treated using primary PCI, low eGFR was associated with increased mortality at the 1-year follow-up. These worse outcomes in patients with low eGFR may be explained, at least partially, by the more complex coronary lesions (higher b-SYNTAX score) and less complete revascularization (higher r-SYNTAX score and lower SRI).

## Figures and Tables

**Table 1:** Baseline characteristics.

	CKD n = 40 (%)	Non-CKD n = 41 (%)	*p*
Age (SD)	70.4 (11.27)	59.8 (13.7)	<0.001
Male	29 (73.5)	30 (72.5)	1
Diabetes	18 (45)	9 (22)	0.035
Smoker (or Former)	20 (50)	22 (53)	0.825
HTA	29 (72.5)	21 (51.2)	0.068
Hypercolesterolemia	29 (72.5)	21 (51.2)	0.068
Prior MI	13 (32.5)	2 (4.9)	0.002
Prior PCI	4 (10)	2 (4.9)	0.432
EF < 30%	22 (55)	10 (24.4)	0.005
Cardiogenic Shock	24 (60)	16 (39)	0.059
CPR	13 (32.5)	15 (36.6)	0.816

CKD—chronic kidney disease; HTA—arterial hypertension; MI—myocardial infarction; PCI—percutaneous coronary intervention; EF—ejection fraction; CPR—cardio-pulmonary resuscitation; SD—standard deviation.

**Table 2:** Angiographic and procedural characteristics. Values are mean ± standard deviation, median (inter-quartile range), or number (%).

	CKD n = 40 (%)	Non-CKD n = 41 (%)	*p*
TIMI 0/1 flow	7 (17.5)	12 (29.3)	0.211
LM collaterals in TIMI 0/1	2 (28.6)	8 (66.7)	0.109
Multivessel Disease	35 (87.5)	21 (51.2)	<0.001
bSYNTAX	30.5 (14.25)	23 (18.5)	0.015
rSYNTAX	8.5 (10.8)	0 (3.5)	<0.001
SRI	67.8 (34.6)	100 (12.5)	<0.001
LM Bifurcation	28 (70)	23 (56.1)	0.195
Two-Stents Technique	5 (12.5)	2 (4.9)	0.084
No. of Stents (SD)	1.8 (1.1)	1.4 (0.6)	0.066
Final Diameter (mm) (SD)	3.86 (0.45)	3.95 (0.53)	0.423
Multivessel PCI	15 (37.5)	13 (31.7)	0.584

CKD—chronic kidney disease; TIMI—thrombolysis in myocardial infarction; LM—left main; b/rSYNTAX–baseline/residual SYNTAX score; SRI—SYNTAX revascularization index; PCI—percutaneous coronary intervention; SD—standard deviation.

**Table 3:** Univariable and multivariable logistic analysis for the prediction of 1-year all-cause mortality. Model I: estimated glomerular filtration rate adjusted for cardiogenic shock and TIMI flow.

Variables	Univariate Analysis			Multivariate Analysis		
	OR	95% CI	*p*	OR	95% CI	*p*
Cardiogenic Shock	5.75	2.19–15.10	<0.001	3.48	1.22–9.92	0.020
TIMI 3/2 vs. 0/1	0.27	0.091–0.81	0.020	0.22	0.06–0.83	0.025
eGFR	0.97	0.95–0.99	0.005	0.97	0.96–0.99	0.021

TIMI—thrombolysis in myocardial infarction; eGFR—estimated glomerular filtration rate; OR—odds ratio; CI—confidence interval.

**Table 4:** Univariable and multivariable logistic analysis for the prediction of 1-year all-cause mortality. Model II: estimated glomerular filtration rate (eGFR) adjusted for left ventricle low ejection fraction (<30%), diabetes mellitus, and prior MI.

Variables	Univariate Analysis			Multivariate Analysis		
	OR	95% CI	*p*	OR	95% CI	*p*
Left Ventricle EF < 30%	2.75	1.09–6.89	0.031	1.99	0.74–5.33	0.168
DM	1.25	0.49–3.16	0.6	0.81	0.28–2.31	0.694
Prior MI	2.16	0.69–6.80	0.18	1.18	0.33–4.19	0.796
eGFR	0.97	0.95–0.99	0.005	0.97	0.96–0.99	0.027

EF—ejection fraction; DM—diabetes mellitus; MI—myocardial infarction; eGFR—estimated glomerular filtration rate; OR—odds ratio; CI—confidence interval.
